# Optimizing Treatment for Depression in Primary Care Using Psychotherapy Versus Antidepressant Medication in a Low-Resource Setting: Protocol for the OptimizeD Randomized Controlled Trial

**DOI:** 10.21203/rs.3.rs-6716211/v1

**Published:** 2025-05-23

**Authors:** Julia R. Pozuelo, Anuja Lahiri, Rahul S.P. Singh, Arvind Kushwah, Mimansa Khanduri, Akanksha Shukla, Azaz Khan, Sruthi G., Varun Shende, Yashika Parashar, Yashwant K. Mehra, Anant Bhan, Ronald C. Kessler, Daisy R. Singla, John A. Naslund, Karmel Choi, Pim Cuijpers, Robert DeRubeis, Mohammad Herzallah, Chunling Lu, Jordan W. Smoller, Tyler J. VanderWeele, Abhijit Rozatkar, Michelle Melwyn Joel, Debasis Biswas, Shubham Atal, Umay Kulsum, Steven D. Hollon, Vikram Patel

## Abstract

**Background:** Psychotherapy and antidepressant medications are first-line treatments for depression, and they both have significant treatment effects on average. However, treatment response varies widely across patients, and neither approach is universally effective. Identifying the most effective treatment for each patient is critical everywhere, but particularly in low-resource settings where access to mental health care is limited. The Optimizing Depression (OptimizeD) trial aims to explore whether different patients respond differently to behavioral activation therapy versus antidepressant medication and if providing each patient with their optimal treatment improves outcomes in primary care. **Methods:** We plan to randomize 1,500 patients with moderate to severe depression (defined as a Patient Health Questionnaire [PHQ-9] score ≥10) from primary healthcare settings in Bhopal, India, with equal allocation either to a culturally adapted behavioral activation therapy delivered by trained counselors (Healthy Activity Program) or to antidepressant medication (fluoxetine). Treatment will last 3 months, with remission (defined as PHQ-9 score <5) at 3 months as the primary endpoint. Using machine learning, we will attempt to develop a precision treatment rule that leverages baseline clinical, psychological, cognitive, socioeconomic, and biological data to predict which treatment is most likely to achieve remission for each patient. Cost-effectiveness analysis will then assess whether the added costs of optimizing treatment are justified by improvements in remission, recovery, and cost savings at the health system and societal levels. Secondary and exploratory objectives include assessing the effectiveness of optimization in a range of secondary outcomes, evaluating treatment mechanisms, and exploring whether incorporating genetic and biological markers as predictors improves treatment optimization. **Discussion:** The OptimizeD trial will evaluate whether baseline information collected in routine care can inform optimal depression treatment selection and identify predictors of nonresponse to facilitate timely specialist referrals. Findings have the potential to enhance personalized depression care in primary health systems, particularly in low-resource settings, with broader implications for global public health. **Trial registration:** ClinicalTrials.gov (NCT05944926; registered July 2, 2023) and Clinical Trials Registry India (CTRI/2024/01/061932; registered January 29, 2024).

## 1. BACKGROUND

Depression is a common mental disorder and a leading cause of disability (1, 2). The burden is particularly pronounced in low-resource settings, where access to mental health services remains limited (3, 4). Primary care facilities often serve as the first—and frequently only—point of contact for individuals with depression, yet the condition remains vastly underdetected and undertreated (5). This treatment gap contributes to adverse social, economic, and health outcomes, with far-reaching implications for individuals, families, and society as a whole (6–10).

Depression is treatable, and the World Health Organization recommends two first-line treatments for moderate to severe depression in primary care: (1) brief psychological therapy (such as behavioral activation) and (2) generic antidepressant medications (such as fluoxetine) (11, 12). Meta-analyses support these recommendations, showing that both have significant treatment effects on average (13, 14).

Despite the availability of effective interventions, two fundamental challenges complicate efficient resource allocation (15). First, individual responses vary significantly, with fewer than 25% of patients achieving remission with initial treatment (16–20). This heterogeneity in response makes it difficult to determine the most suitable option for each patient without relying on trial and error. Second, more than half of patients may not respond to either treatment, yet identifying these refractory cases early to facilitate timely referral to specialist care remains complex (21, 22). Consequently, many patients whose initial treatment fails never proceed to more effective interventions, increasing the risk of chronicity and prolonged impairment (23). These challenges are particularly acute in low-resource settings, in which ineffective first-line treatment carries high opportunity costs and diverts scarce mental health resources from more effective uses (3, 4, 24).

Precision mental health has emerged as a promising approach to addressing these challenges by identifying patient characteristics associated with differential treatment response and developing algorithms to guide personalized treatment selection and referral pathways (25–27). Early research in this domain focused on individual predictors of treatment response, such as depression subtype or severity, but no single indicator has proven adequate to inform clinical decisions meaningfully (17, 28, 29). Recent advances in statistical methodology and machine learning now allow researchers to process numerous patient variables simultaneously, developing personalized prediction algorithms that improve allocation accuracy (17, 25, 30–35). Such approaches can be especially valuable in settings with limited resources, in which precision treatment rules may inform not only which treatment to offer but also who should be prioritized for treatment when resources are limited.

Despite these promising developments, several gaps remain. First, most studies have small sample sizes, limiting power to detect moderation and reducing prediction accuracy (25, 36). Second, many studies assess only a limited set of characteristics, making it difficult to identify which factors predict differential treatment response, highlighting the need for broader baseline assessments that can later be refined (17). Third, most precision treatment research has been conducted in specialized mental health facilities in high-income settings, limiting its relevance to low-resource primary care (37, 38). Fourth, many existing precision approaches rely on advanced diagnostic technologies (e.g., genetic markers, neuroimaging) that are impractical for widespread implementation in low-resource primary care settings (39, 40).

#### Study objectives

The Optimizing Depression (OptimizeD) trial aims to address these gaps by developing and validating a precision treatment approach for depression in primary care settings in India. The study will randomize

1,500 patients with moderate to severe depression to receive either a culturally-adapted behavioral activation psychotherapy (Healthy Activity Program, HAP) (41, 42) or antidepressant medication (ADM, fluoxetine). We will then use machine learning methods to investigate whether a useful precision treatment rule (PTR) can be developed to predict differential treatment response based on baseline patient characteristics.

The OptimizeD trial explores several novel innovations. Unlike many precision mental health studies that rely on complex biomarkers or expensive neuroimaging techniques, we focus on self-reported measures that are simple, scalable, and feasible to implement in routine primary care. These include variables spanning multiple domains—clinical, psychological, cognitive, socioeconomic, and biological—reflecting depression's multifaceted nature (43). Each baseline variable being considered meets three essential criteria: (1) prior evidence or theory suggesting that the variable might predict differential treatment outcome; (2) cross-cultural applicability; and (3) feasibility of collection in primary care settings. With a sample of 1,500 participants, the study is well-powered to detect meaningful treatment moderators and validate findings in an independent subsample (36). Beyond developing the PTR, we will identify baseline predictors of treatment nonresponse to guide specialist referrals and evaluate the cost-effectiveness of optimal versus non-optimal treatment assignment. The inclusion of long-term follow-up will determine whether treatment assignment to optimize 3-month outcomes also improves sustained recovery. Additionally, two exploratory aims will extend the study's impact: investigating potential treatment mechanisms through mediation analysis and examining whether biological markers can enhance prediction accuracy when added to the PTR.

Overall, the OptimizeD trial will serve as a proof-of-concept for applying precision mental health principles in primary care settings in low-resource environments. Success in developing a scalable, data-driven approach to treatment selection would have the potential to shift practice in mental health care away from a "one-size-fits-all" trial-and-error approach to a more personalized, evidence-based approach that optimizes outcomes while maximizing efficiency.

The overarching goal of this study is to improve depression outcomes in primary care by determining the preferred treatment between two practical alternatives for each patient. The primary objectives focus on assessing the effectiveness of optimizing treatment in achieving remission and evaluating cost-effectiveness at 12 weeks. Secondary objectives evaluate the effectiveness of optimization in improving secondary outcomes. Exploratory objectives include examining potential mediators and assessing biological predictors to enhance treatment prediction (Table 1).

[INSERT Table 1]

## 2. METHODS

#### 2.1 Trial design

The OptimizeD trial is a phase III, single-blind, two-arm, parallel-group randomized precision trial. Eligible participants will be randomized to either treatment modality (HAP vs. ADM) in a 1:1 ratio. The protocol conforms to the Standard Protocol Items: Recommendations for Interventional Trials (SPIRIT 2025) guidelines (44) (see checklist in Table A1 in the appendix).

### 2.2 Study setting

The study is currently being implemented in Bhopal district, located in Madhya Pradesh, a state in central India with a population of over 87 million, of whom nearly 73% reside in rural areas (45,46). This state ranks among the lowest on the Human Development Index and last on the Global Hunger Index among all Indian states (47,48).

The recruitment sites comprise 8 – 14 public sector Primary Healthcare Facilities in Bhopal, selected based on a set of predefined criteria: availability of a medical officer authorized to prescribe antidepressants, presence of a pharmacist, proximity to the laboratory for biological specimen storage, and availability of a private room for assessments. Centers meeting the highest number of criteria were prioritized for inclusion. Prior research in similar facilities identified a 17% point prevalence of moderate to severe depression (49,50).

### 2.3 Eligibility criteria

Inclusion criteria for this trial are: (1) Provision of signed and dated informed consent form; (2) Age 18 or above; (3) Attend one of the selected Primary Health Care Facilities in the study setting; and (4) A score of 10 or above on the Patient Health Questionnaire-9 (PHQ-9) (51). Exclusion criteria of this trial are: (1) Women who are pregnant or are breastfeeding or lactating; (2) Patients with a history of psychosis, including schizophrenia spectrum disorders or bipolar disorder; (3) Patients planning to permanently move out of the study area during the follow-up period; (4) Patients with evidence of cognitive impairment based on the Mini-Mental State Examination-2 (MMSE-2 Brief Version) (52); (5) Patients who do not speak either English or Hindi (the latter being the primary language of the region); (6) Patients undergoing treatment for depression at the time of recruitment or who completed treatment within one month prior to recruitment; (7) Patients at imminent risk for suicide as determined by the Columbia Suicide Severity Rating Scale (C-SSRS) and the assessment of the trial psychologist and/or trial psychiatrist (53); and (8) Patients from households in which another member has been recruited into the study. There are no exclusions for other nonpsychotic comorbidities or other medical conditions, and no exclusions based on race, ethnicity, caste, or religion.

### 2.4 Study procedures

Recruitment is conducted by trained and supervised Research Assistants (RAs) who approach individuals attending primary healthcare facilities. Potential participants are introduced to the study and invited to complete an eligibility survey administered electronically via tablet using Research Electronic Data Capture (REDCap) (54). The survey follows a stepwise format, beginning with the PHQ-2 and expanding to the full PHQ-9 for those scoring ≥1. Individuals who endorse the 9th item of the PHQ-9 (score≥2) complete the C-SSRS (53), and those identified as high-risk are referred to the trial psychologist and/or trial psychiatrist. Patients determined to be at imminent risk for suicide (e.g., those requiring hospitalization) are excluded from the study. If not deemed at imminent risk and otherwise eligible, the participant may proceed with recruitment. Additional questions assess exclusion criteria such as psychiatric history and plans to relocate, among others.

Those who meet all eligibility criteria and provide consent are invited to complete the baseline assessment, which includes questionnaires, neurocognitive tasks, and biospecimen collection. The assessment covers multiple domains, including clinical (e.g., depression severity, comorbidities), psychological (e.g., personality traits), cognitive (e.g., attention, memory), socioeconomic (e.g., education, income), and biological (e.g., genetic and inflammatory markers). The full assessment schedule is detailed in Table 2. The OptimizeD baseline battery was developed through a systematic, multimethod process aimed at balancing breadth and brevity. The process involved updating a systematic review to identify constructs associated with differential treatment response, conducting an expert survey to refine the list, and selecting culturally appropriate and scalable measurement tools. The initial battery was piloted with 200 participants, and data from the pilot were used to reduce redundancy and streamline the assessment through exploratory factor analysis and machine learning techniques. This optimization reduced administration time by 27% while retaining key constructs. Table A2 presents the final set of measures included in the baseline battery. A detailed description of the development process will be provided in a separate publication.

To explore whether genetic and biomarker data can improve treatment response prediction, participants are also invited to provide a 5 mL intravenous blood sample for genetic/biomarker analysis. Participation in the blood draw is optional and not required for inclusion in the trial. For those unwilling or unable to provide a blood sample, a 1 mL saliva sample is requested as a non-invasive alternative.

Immediately following this assessment, participants are randomized to one of the two treatment arms. Study assessments occur at weeks 1.5, 6, and 12, with follow-up assessments at 6, 9, and 12 months. Primary and secondary outcomes – remission, cost-effectiveness, severity, anxiety, disability, patient-rated change, and well-being – are assessed at 12 weeks. Recovery is assessed at the 12-month follow-up. Measurement tools are detailed in Tables 1 and A2. All study assessments, except for the baseline assessment, are conducted by outcome assessors. Suicidal behavior is assessed at all time points, and those identified as high risk are referred to the trial psychologist/psychiatrist for further evaluation. The trial design is presented in Figure 1.

[INSERT TABLE 2 AND FIGURE 1]

### 2.5 Randomization and blinding

Participants are randomized in a 1:1 ratio to either HAP or ADM using a computerized algorithm developed by the Centre for Healthcare Randomised Trials (CHaRT) at the University of Aberdeen (55), an organization independent of the OptimizeD trial team. The algorithm employs a maximum tolerated imbalance (MTI) procedure with a sliding window approach, incorporating a random element to minimize allocation predictability (56,57). This dynamic method balances key prognostic factors (clinic, sex, and PHQ-9 score <15) while reducing determinism in treatment assignment. Participants are allocated using CHaRT's online software, with the Trial Manager overseeing enrolment per assignment.

Although the main purpose of the trial is to compare optimal vs. non-optimal treatment rather than HAP vs. ADM, the outcome assessors will remain blinded to treatment allocation to improve methodological rigor. Participants and treatment providers are aware of treatment allocation, but participants are asked not to disclose their treatment to assessors. If an outcome assessor is unintentionally unblinded, the participant will be reassigned to another blinded assessor, and the incident will be documented for sensitivity analysis. Assessments are conducted at the participants' homes, clinics, by phone, or at a convenient location (e.g., parks, temples).

### 2.6 Interventions

#### 2.6.1 Healthy Activity Program (HAP)

HAP is a brief, psychological treatment based on behavioral activation, an empirically supported treatment recommended by the WHO (11). HAP incorporates strategies such as problem-solving, assertiveness training, and activation of social networks in a person-centered way. In our original HAP trial, 64% of participants with moderately severe to severe depression achieved remission (PHQ score<10) at 3 months, compared to 39% in the control group receiving enhanced usual care (41). These benefits were sustained at 12 months (42).

HAP is delivered over 6-8 sessions over a 3-month period by trained counselors with undergraduate/graduate degrees in psychology, social work, or other related fields, but without prior counseling experience. HAP is delivered in an individual format, with each session lasting approximately 30–40 minutes. Sessions are conducted face-to-face, at the participant's home, or at a convenient location of the participant's choosing. If in-person sessions are not feasible, these may be conducted by phone.

HAP is a manualized treatment that is structured into three phases: (1) *Beginning phase (1–2 sessions):* Introduction to treatment, fostering hope, and building rapport; (2) *Middle phase (3–6 sessions)*: Core intervention strategies, including activation, problem-solving, and assertiveness skills, with relapse prevention as needed. Additional sessions (up to two) may be added for patients with persistently high PHQ-9 scores (PHQ ≥ 10) and limited activation (measured by the number of homework assignments not completed, partially completed, or fully completed); and (3) *Ending phase (1 session):* Reviewing gains, consolidating skills, and preparing for termination. Each of these phases is characterized by specific goals and activities collaboratively agreed upon by the counselor and participant. The number of sessions may vary between 6 and 8, contingent upon the patient's symptomatic response (as indicated by PHQ-9 scores). Patients who achieve a PHQ-9 score≤5 in sessions 3 or 4 and express interest in discharge may be considered for early termination after the 4th session, following a review with the HAP team leader. Further details of HAP can be found in the HAP manual (58).

#### 2.6.2 Antidepressant medication (ADM)

Participants in the ADM group will receive fluoxetine, a selective serotonin reuptake inhibitor (SSRI) widely recognized for its safety and efficacy in treating depression (59). Fluoxetine is part of India's Essential Drug List (60) and has demonstrated effectiveness in primary care (61). A placebo-controlled trial in India reported a remission rate of 70% at 2 months among patients treated with fluoxetine, compared to 54% in the placebo group (61), which is notably higher than the range typically observed in meta-analyses (59).

The acute treatment phase will be delivered for 3 months, with clinicians conducting follow-up sessions at weeks 3, 6, and 12 to assess tolerability and symptoms and determine dosage adjustments. Participants will start at a dose of 20 mg per day and can be escalated to higher doses (40 mg) at subsequent visits, depending on tolerability and symptoms. The dose may be dropped to 10 mg daily if side effects occur.

During the continuation phase (months 4–12), the dose will be maintained based on PHQ-9 scores and tolerability, with adjustments made in consultation with the Medical Officer or the trial psychiatrist. At the 12-month visit, participants on doses higher than 20 mg/day will have their dose tapered to 20 mg/day for two weeks before discontinuation, while those on 20 mg/day will discontinue directly.

If fluoxetine is not tolerated, clinicians may switch participants to escitalopram at week 6, another SSRI recognized for its safety and efficacy, and also included in India's National List of Essential Medications (60,62). Escitalopram treatment will be initiated at 10 mg per day, with the option to increase to 20 mg daily based on tolerance and treatment response. These participants will undergo an additional follow-up session at week 9 to monitor progress.

If a participant experiences a serious side effect from taking fluoxetine or escitalopram, they will be referred to specialist care. Outcome data for participants switched to escitalopram or referred to specialist care will continue to be collected, and we will conduct sensitivity analyses to assess the impact of these treatment modifications on primary and secondary outcomes.

Medical Officers (MOs) from the recruiting clinics are the primary healthcare providers prescribing medication in OptimizeD. In situations in which the MO is not available, the trial psychiatrist has been authorized by the state health system to prescribe medication via telemedicine. Both fluoxetine and escitalopram are provided free of charge to participants through government supply channels.

#### 2.6.3 Quality and fidelity of the intervention

##### 2.6.3.1 HAP

HAP counselors participate in weekly group supervision and monthly individual supervision to review audio-recorded sessions, practice role-plays, and address patient-specific challenges. Group supervision, facilitated by a HAP supervisor, is conducted in peer groups of 5–6 counselors, while individual supervision, led by the HAP team leader, provides more in-depth, one-on-one guidance.

We assess HAP quality using the following metrics:

- *Participants' adherence to HAP treatment.* We will explore several indicators, such as treatment dose (measured by the number of sessions completed) and homework engagement. Treatment compliance will be defined as attending at least six sessions or requesting discharge after four sessions with PHQ-9 scores below 5 in both the third and fourth sessions.
- *Therapy quality.* We will assess the quality of two agents:
    - Quality of HAP counselors: Assessed through the rating of audio-taped sessions using a specifically developed scale called the Quality of Behavioral Activation Scale (Q-BAS), a 20-item, 5-point Likert scale (63,64). Higher scores indicate higher implementation quality. Weekly supervision sessions include independent ratings of audio-recorded sessions by the counselor, their peers, and the supervisor, with aggregate scores calculated for each group.
    - Quality of the HAP supervisor: Assessed using the EMPOWERS rating scale (a five-point Likert scale with 8 items that assesses the skills required to facilitate supervision sessions) (64). At the end of the session, all the HAP counselors and the HAP supervisor will rate the facilitation quality of the session. Aggregate scores of all counselors and the supervisor will be evaluated.

We will assess HAP fidelity using the following approach:

- *External fidelity rating.* Independent experts, unaffiliated with the OptimizeD trial but previously involved in HAP development or delivery, will randomly evaluate and rate 5% of all audio-recorded HAP sessions, stratified by counselor and treatment phase. Each week, the same audio-recorded sessions that are rated by counselors and the supervisor are also assessed by fidelity assessors to ensure consistency in scoring.

##### 2.6.3.2 ADM

We will assess ADM quality using the following metrics:

- *Participants' ADM adherence.* We will explore several indicators, such as appointment attendance and dose adherence. Pill counts will be conducted at regular intervals to measure medication adherence. At each visit, participants will receive three sequentially numbered blister packs, each containing 10 pills. Participants will be asked to retain any empty blisters of medications and hand them over to the research staff at the next visit. The number of blisters and pills returned will be counted and recorded. To calculate adherence, we will sum the total number of pills and divide it by the days in treatment.

- *Quality of ADM delivery.* We will assess the following indicators:
  - Medication dispensation: We will assess the fidelity of antidepressant delivery by monitoring the accurate dispensation of medications to participants by RAs. This will involve tracking the prescription and distribution of antidepressant medications according to established treatment protocols.
  - Medical Officer training: We will measure whether the MO attended the training on the OptimizeD trial and clinical protocol.
  - Adverse event monitoring: Adverse events or side effects experienced by participants while taking antidepressant medications will be monitored and reported, ensuring they are appropriately managed.

Unlike psychotherapy interventions, for which external ratings by independent experts are commonly employed as a measure of fidelity, administering antidepressant medications does not lend itself to this type of external rating. Instead, we will focus on assessing the quality of ADM delivery to ensure the safe and effective administration of medication within the study.

#### 2.6.4 Strategies to improve treatment adherence

Several strategies will be employed to enhance treatment completion rates, drawing on our experience with previous studies in the setting (41,61,65). First, participants will receive thorough education about their treatment before providing consent, including the purpose, potential benefits, and possible side effects of each intervention. Second, MOs will play a key role in reinforcing treatment adherence. Third, and to ensure continuity of care, the trial psychiatrist is authorized by the state health system to prescribe antidepressant medications via telemedicine in cases where the MO is unavailable. Fourth, HAP sessions will be delivered flexibly, with options for participants to attend sessions at clinics, their homes, or other preferred locations. The HAP counselors will accommodate participants' schedules, including after-work hours and weekends, and offer telephonic sessions for those uncomfortable with in-person meetings. Fifth, medications will be delivered to participants' homes or other convenient locations whenever they are unable to visit the clinic, minimizing logistical barriers. Sixth, family involvement will also be encouraged, with participant consent, to provide additional support for treatment adherence. Seventh, reminder calls will be made 48 hours before scheduled treatment sessions, followed by text message reminders 24 hours prior. If participants miss a scheduled session, the research team will reach out to determine the reason and reschedule to maintain engagement and continuity in the treatment process. These strategies collectively aim to reduce barriers to adherence and enhance treatment outcomes.

#### 2.1.1 Criteria for discontinuing or modifying allocated interventions

There are several circumstances that might result in a participant discontinuing treatment, as outlined in Table 3. If a participant discontinues HAP or ADM, we will nonetheless attempt to keep them in the study in terms of completing follow-up assessments.

[INSERT TABLE 3]

#### 2.1.1 Participant discontinuation/withdrawal

Participants may withdraw from the study at any time upon request. Additionally, investigators may discontinue participants due to being lost to follow-up or if a medical condition arises that could compromise participant safety. For participants who become pregnant, those in the HAP arm will continue their sessions without modification, while those in the ADM arm will undergo a treatment review with the clinical team to reassess medication safety and adjust the treatment plan as needed. Withdrawal reasons will be recorded, and once randomized, participants will not be replaced. Lost-to-follow-up will be determined after three unsuccessful contact attempts, including phone calls and a home visit, with all contact attempts documented. Participants who explicitly choose to withdraw or remain unreachable after these attempts will be classified as withdrawn. Participants who wish to rejoin the study may resume from their original enrollment point.

### 2.1 Strategies to improve adherence to study assessments

Several strategies will be employed to promote adherence to research assessments. First, participants will receive clear information about the study's time commitments before enrollment to ensure informed consent. Second, Medical Officers will encourage participation at study onset and will intervene if non-adherence is observed. Third, surveys will be divided into shorter segments to reduce participant burden, with breaks included to maintain engagement. Fourth, while participants do not receive direct payment for participation, they will receive 500 INR for each of the seven scheduled assessments, totaling 3,500 INR (approximately USD 40). Fifth, reminder calls and text messages will be sent 48 and 24 hours before each scheduled study assessment to minimize missed visits. Finally, if a participant misses a scheduled session, the research team will contact them to identify and address barriers, rescheduling the session as needed.

### 2.2 Relevant concomitant care permitted or prohibited during the trial

Participants receiving any treatments for depression (psychological or pharmacological) at the start of the trial or within one month prior to recruitment will not be eligible for participation. Participants may use analgesics for pain control, including over-the-counter medications such as non-steroidal anti-inflammatory drugs (e.g., ibuprofen, aspirin) and paracetamol, as well as prescribed medications for conditions other than depression. Medication usage will be assessed at baseline, 3-month, and 12-month study assessments.

### 2.3 Provisions for post-trial care

At the 12-month assessment, which marks the final study assessment, participants will be briefed on their current symptom levels of depression. Those with PHQ-9 scores≥10, as well as those scoring between 5–9 who show less than 50% symptom improvement from baseline, will be offered the option of being referred to specialist care at the collaborating tertiary care institute – the All India Institute of Medical Sciences (AIIMS Bhopal) for further mental health support services.

#### 2.4 Sample size

This study is designed to provide sufficient power to generate and validate the PTR to predict differential responses to HAP versus ADM (primary objective 1). A total of 1,500 participants will be randomized to either HAP or ADM. Of these, the first 1,000 participants will be used to train the PTR algorithm, using cross-validation for hyperparameter tuning and nested cross-validation for calibration, while the remaining 500 participants will form an independent validation sample to evaluate the algorithm's predictive accuracy. The sample size required for generating a valid PTR depends on the anticipated attained marginal improvement (AMI), which quantifies the additional benefit of assigning patients to their optimal treatment compared to the intervention with the larger average treatment effect. Power calculations based on simulations using realistic assumptions about underlying processes generating the AMIs suggest that 300-500 patients per arm (600-1,000 total) are required to detect AMIs in the range from 5% (minimal improvement) to 25% (substantial improvement) with statistical power of at least 0.8 using two-sided p=0.05 significance tests (36). Our trial, with 750 participants per arm, will consequently be well powered to develop a useful PTS if meaningful heterogeneity exists in differential treatment response and our baseline covariates are sufficiently predictive to find AMI in this range. The economic analyses (primary objective 2) will utilize the full sample of 1,500 participants. This ensures adequate power to perform cost-effectiveness comparisons between HAP and ADM.

While mediation analyses (exploratory objective 1) are not the primary focus of the sample size determination, the total sample size of 1,500 is approximately three times larger than the 500 participants typically needed to detect mediation effects with adequate power (66). However, detecting moderated mediation effects is more challenging, often requiring four times the sample size used for standard mediation analyses (67). Applying this rule of thumb, our sample size of 1,500 aligns with the recommended threshold for detecting moderated mediation effects, though power will depend on the effect sizes of both the direct and indirect effects (68). The genetic analyses (exploratory objective 2) are intended to be exploratory, with power calculations currently premature, particularly in a cross-ancestry context. These analyses will explore whether integrating polygenic risk scores and other genetic and biological biomarkers can enhance the predictive utility of the PTR.

In summary, this study is powered to generate and test a robust PTR (primary objective 1), conduct adequately powered economic analyses (primary objective 2), and provide exploratory insights into mediation and genetic moderators to guide future research.

#### 2.5 Data management

Data will be primarily collected using REDCap (54) via tablets and uploaded daily to Sangath's secure server. Neurocognitive tasks will be administered via tablets using NubiS, a software designed for complex cognitive assessments (69). HAP-related data will be collected using the PEERS app, a smartphone-based system built on the CommCare platform (70). For participants in the ADM arm, pill counts will be recorded on paper and entered into RedCap. Additionally, audio recordings of surveys will be collected from participants who consent to this component to facilitate data quality checks. Paper-based documents, such as signed consent forms and patient cards, will be securely stored in locked filing cabinets at the Sangath Bhopal office. Data will be regularly backed up, de-identified, cleaned, and stored securely for a minimum of seven years post study completion. All identifiable information will be stored separately from the research data, and participants will be identified in study files only by their unique study ID.

The team at AIIMS Bhopal will manage the processing of the biological samples. Samples will be stored in temperature-controlled, access-restricted facilities and labeled with study IDs only. De-identified genetic data will be transferred via secure, encrypted channels to Harvard/Massachusetts General Hospital for analysis. Biospecimens will be retained in accordance with local regulations and participant consent.

### 2.6 Statistical analysis

#### 2.6.1 Precision treatment rule (PTR)

We aim to develop a precision treatment rule (PTR) to identify the optimal treatment for each patient by modeling how baseline characteristics interact with treatment type to predict outcomes. This will involve estimating predicted outcomes for each patient under both treatment conditions (HAP and ADM) and calculating an individual-level difference score to identify optimal treatment assignment.

Traditional regression-based PTR models typically rely on correctly specifying main effects and simple two-way interactions, where treatment effects vary based on specific baseline characteristics but do not account for more complex interaction patterns (71). For example, treatment effects might be modeled as a function of treatment assignment (T), gender (G), and baseline depression severity (BDS) as follows:

$\text{Outcome} = b_0 + b_1T + b_2G + b_3BDS + b_4(T \times G) + b_5(T \times BDS)$,

However, interactions can be far more complex, involving nonlinearities and higher-order effects. For example, the differential effect of treatments A and B among women compared to men might only be present at specific levels of baseline depression severity. To account for these complexities, we will use a more flexible and robust machine learning approach that estimates interactions based on counterfactual logic. In this approach, prediction models are estimated separately within each treatment arm, assigning each patient two predicted outcome scores (one for HAP and one for ADM) based on baseline covariates. These scores represent the expected outcome under each treatment, allowing us to estimate how much a patient’s outcome would differ if they had been assigned to one treatment versus the other. This difference is known as the conditional average treatment effect (CATE).

The within-arm models will be estimated using the Super Learner (SL) algorithm, an ensemble machine learning method that allows for both nonlinearities and higher-order interactions (72). The optimal combination of algorithms is defined based on an objective function, which in our case will be maximum reduction in cross-validated mean-squared error in the outcome. SL improves accuracy and reduces the risk of model misspecification (73). Our SL library will include a range of modern machine learning algorithms such as penalized regression, tree-based methods, support vector machines, and gradient boosting. Importantly, preliminary models will be estimated in a nested cross-validation framework to predict differential engagement with HAP and differential adherence to ADM. This step allows us to create composite predictors to determine the extent to which the PTR is predicting differential engagement/adherence versus differential intervention effectiveness (74).

Once the within-arm prediction models are created, we will assign predicted probabilities to each patient based on each equation. Since the two subsamples of patients are equivalent by design, both equations should apply to both subsamples. The difference between these two predicted scores at the individual level will represent the estimated CATE, indicating the expected impact of assigning a patient to HAP versus ADM.

These initial CATE estimates will be used as the outcome in a second SL ensemble analysis to identify baseline predictors of treatment effect differences. Unlike traditional approaches, this method does not require accurate specification of main effects because composite interactions – represented by CATEs –are being directly estimated by pooling estimates across algorithms in the ensemble. Doubly robust methods will be applied in this analysis to adjust for potential loss to follow-up.

If CATE estimates differ significantly across patients, with some estimates favoring HAP and others favoring ADM, we will use nested cross-validation to estimate the expected improvement in aggregate outcomes if patients were assigned to treatments based on their estimated CATEs rather than at random. Additionally, if one treatment is found to be superior overall, we will also estimate the expected improvement in aggregate outcomes if patients were assigned to treatments based on their estimated CATEs rather than assigning all patients to the treatment with the best average effect.

A key challenge in using complex machine learning methods is the risk of overfitting, which can degrade model performance when the model is applied elsewhere. To address this, we will implement several strategies. First, we will exclude potential predictors with fewer than 10 observations in the smaller cell of a dichotomous variable. Second, we will use cross-validation to assess whether potential predictors have stable, significant univariable associations with the outcome. Third, associations among potential predictors that pass the second test will be examined, and only predictors with correlations of $r \geq 0.80$ will be retained in the final predictor set. Fourth, potential predictor screening and restriction will be used at the analysis stage to avoid overfitting by allowing only 5%, 10%, or 20% as many predictors in the model as we have patients with outcomes to predict. Selection of this restricted number of predictors will be carried out using Lasso penalized regression for linear algorithms and random forest for more complex algorithms. A separate selection procedure will be used for each fold in the 10-fold cross-validation used to estimate models. Fifth, each classifier will be included in the ensemble multiple times, each time with a distinct set of hyperparameter values selected at random across a multivariable grid of key features. The SL weighting procedure will then be used to select the optimal hyperparameter configuration to include in the ensemble. Lastly, the final SL ensemble will consist of three times the number of classifiers, each estimated with distinct predictor sets within each cross-validation fold.

#### 2.6.2 Cost-effectiveness analysis

Our economic evaluation will assess the cost-effectiveness of using a PTR to guide depression treatment by comparing the costs and outcomes of optimal versus non-optimal treatment assignments. We will examine (1) total costs and cost per remission or recovery for each group; (2) the cost-effectiveness of random assignment to optimal vs. non-optimal treatment; and (3) how cost-effectiveness varies across different levels of predicted probability of remission and recovery from the PTR. We hypothesize that optimal treatment will be more cost-effective than non-optimal treatment in achieving remission or recovery, and that cost-effectiveness will increase with greater predicted benefit, as indicated by higher PTR differential scores.

Costs will be assessed from both the health system and societal perspectives, including direct, indirect, and opportunity costs. System-level costs will be estimated using instruments based on the WHO health systems building blocks framework,(75,76) capturing resources used for ADM and HAP training and delivery, biological collection and analysis, and PTR measurement. Cost components will include staff time, infrastructure, equipment, and unpaid contributions. Patient-level costs will include the costs of participating in treatment, such as transportation and lost wages, captured with a tailored 7-item tool. We will also collect participants' out-of-pocket and non-medical costs data of obtaining general care using the Client Service Receipt Inventory (CSRI) (77). The CSRI data will allow us to (1) examine the difference in changes of household economic burden resulting from receiving general care between the optimized and non-optimized groups, and (2) estimate the difference in savings at the health system level resulting from treating depression using optimized versus non-optimized methods. Effectiveness will be measured by (1) the likelihood of remission and recovery (using multilevel logistic regression) and (2) quality-adjusted life years (QALYs), derived from WHODAS-II scores. Incremental costs and incremental effectiveness will be derived using the multilevel generalized linear models. All models will adjust for clustering at the clinic level.

We will obtain the mean and 95% confidence intervals of the incremental cost-effectiveness ratios using the bootstrapping method (78,79). Cost-effectiveness acceptability curves will be produced across a range of willingness-to-pay thresholds from either a health system perspective or a societal perspective. Finally, we will explore the added value of the PTR by conducting separate analyses for remission and recovery, stratifying participants assigned to their optimal treatment into four groups based on the predicted probability of each outcome (<25%, 25–50%, 51–75%, >75%). This allows us to test whether cost-effectiveness varies with the strength of the PTR recommendation, offering practical guidance for decision-makers on when optimization yields the highest value.

#### 2.6.3 Mediation analysis

Mediation analyses will be conducted using MPlus (version 8.4 or higher) or any other appropriate statistical analysis packages. We will estimate the indirect effects of the proposed mediators on remission and recovery.

We will begin by examining each mediator independently, followed by simultaneous testing of multiple mediators using the Monte Carlo Method for Assessing Mediation. Indirect effects will be estimated as the product of two normally distributed coefficients, and MPlus will be used to simulate the distribution and calculate asymmetric 95% confidence intervals (based on 20,000 repetitions). For analyses involving multiple mediators, we will apply methods from causal mediation analysis to estimate effects through all mediators jointly, modeling both the outcome on all mediators and each mediator separately while adjusting for baseline covariates to control for confounding (80).

#### 2.6.4 Biological Specimen Analysis

At baseline assessment, 5 mL of peripheral blood or 1 mL of saliva will be collected by trained personnel from each consenting participant during their baseline visit. Samples will be transported securely to AIIMS Bhopal and processed for genomic DNA extraction using the QIAamp DNA Blood Kit (81). DNA quantification will be conducted using the Qubit™ Fluorometer (82) with the High Sensitivity (HS) Assay Kit. DNA integrity will be assessed by agarose gel electrophoresis. Purity and concentration will be further confirmed by measuring the 260/280 absorbance ratio (1.8 – 2.0) using a NanoDrop™ spectrophotometer (83,84).

**Genome-wide genotyping will be performed using the Illumina™** Global Screening Array **(GSA), a high-throughput genotyping platform. It employs BeadChip technology and includes more than 650,000 variants across diverse populations, providing coverage of disease-associated and pharmacogenomic markers, and high-value exonic variants** (85)**.**

We will apply standard genomic quality control (QC) and imputation procedures, applicable across population backgrounds. Broadly, variants will be filtered based on sample missingness and other QC criteria, and individuals will also be filtered based on genotype missingness. We will conduct ancestry assignment based on principal components (PC) projected against the 1000 Genomes Project reference panel, followed by additional quality control measures (e.g., for sex mismatch or heterozygosity) within ancestry groups. Imputation will be conducted using standard imputation platforms (e.g., TopMED). Following imputation, additional quality checks will be applied, including the removal of variants with low imputation accuracy and filtering for minor allele frequency and violations of Hardy-Weinberg equilibrium.

##### 2.6.4.1 Polygenic Risk Scores

Polygenic risk scores (PRS) will be calculated as the weighted sum of allele dosages across variants combined with per-variant effects from summary statistics from prior large-scale genome-wide association (GWAS) studies (86). We will generate polygenic scores for major depressive disorder, other psychiatric disorders (e.g., schizophrenia, bipolar disorder, ASD), personality traits (e.g., neuroticism), and cognitive function, in line with existing studies on genetics and treatment response. To improve predictive accuracy, particularly across ancestries, we will use PRS continuous shrinkage (PRS-CS), a Bayesian polygenic scoring method that accounts for linkage disequilibrium using relevant (e.g., South Asian) reference panels from the 1000 Genomes Project (87). PRS-CS has shown superior performance over traditional methods, especially in non-European populations. As methods for optimizing cross-ancestry PRS performance evolve, we will incorporate new approaches as appropriate (88). Using the generated PGS, we will then examine associations between each PRS and PHQ-9 scores at baseline, 12 weeks, and 12 months, as well as remission and recovery, adjusting for principal components, age, and sex. We will also assess whether the inclusion of PRS data improves the predictive accuracy of the personalized treatment rule (PTR) models in identifying optimal treatment assignments.

##### 2.6.4.2 Pharmacogenomic Analysis

CYP2D6 genotyping and metabolizer status analysis will be conducted using TaqMan® CNV(89) and SNP genotyping assays, targeting clinically relevant CYP2D6 alleles. We will determine each participant's metabolizer phenotype as a potential predictor of fluoxetine response. In addition to examining the independent predictive value of CYP2D6 status, we will also assess whether integrating pharmacogenomic data into the PTR models, alongside PRS, improves treatment prediction accuracy.

##### 2.6.4.3 Additional Exploratory Analyses

- *C-Reactive Protein (CRP) measurement*: CRP concentration will be measured in the serum CRP CardioPhase® HS Assay Kit (90) to evaluate the role of systemic inflammation in influencing therapeutic outcomes.
- *Pharmacogenomic Variant Analysis:* We will examine the prevalence of SNPs and CNVs in Tier 1 pharmacogenes (*CYP2D6, CYP2C19, CYP2B6*) related to antidepressants in our central Indian sample and compare them to other Indian and international populations and possible clinical implications.
- *Cross-platform concordance:* As *CYP2D6* CNVs will be assessed using both microarray-based (Global Screening Array) and TaqMan-based genotyping methods, we will evaluate concordance between the two technologies in detecting CNVs.
- *Comparison of DNA Sources:* We will compare genotyping efficiency and quality metrics between saliva-derived and blood-derived DNA samples to assess feasibility for future large-scale studies.

#### 2.7 Oversight and management

The PIs will have overall responsibility for monitoring the integrity of study data and participant safety. In addition, several trial committees have been established to oversee participant safety and data quality, including a Project Management Committee, a Trial Steering Committee, and a Data and Safety

Monitoring Board (DSMB). Details about the roles and composition of these committees are provided in Table 4.

[INSERT TABLE 4]

#### 2.8 Serious adverse events

We define a Serious Adverse Event (SAE) as any adverse event that results in death, is life-threatening, requires hospitalization, causes significant disability, or may jeopardize health and require medical intervention. All SAEs will be assessed for relatedness to study procedures and expectedness by the clinical team. SAEs may be reported during study or intervention visits or via disclosure to research personnel.

SAEs will be compiled by the Trial Manager and shared with the PIs, co-Is, and the Trial Psychiatrist. All SAEs—regardless of relatedness—will be reported to the Sangath IRB within 24 hours of becoming known to the study team. Only SAEs determined to be related to study procedures will be reported to the AIIMS IRB, HMS IRB, the NIMH Program Officer, and the DSMB Chair within two business days of awareness by the Principal Investigators. The Trial Psychiatrist will conduct a follow-up assessment within five business days of the report, and a detailed assessment will be submitted to relevant IRBs and NIMH within 14 days. These procedures are aligned with India's research ethics guidelines to ensure participant safety and regulatory compliance.

## 3. DISCUSSION

Depression represents a significant global health challenge characterized by substantial heterogeneity in treatment response (16–19). This variability presents a critical obstacle for clinicians, particularly in primary care settings with limited resources. Conventional approaches rely on sequential, trial-and-error strategies that often delay remission and increase the risk of chronicity (16, 91). In contexts where accessing treatment is difficult, such stepped-care approaches become impractical. This reality underscores the urgent need to optimize initial treatment selection to improve early outcomes and make the most of limited mental health resources.

The OptimizeD trial aims to address this challenge by developing and validating a pragmatic, precision-guided approach to first-line depression treatment in primary care. By using data that can be feasibly collected in routine practice, the study aims to improve the efficiency and impact of care without relying on specialized technologies or settings. The study's methodological strengths—including its large sample size, rigorous design, long-term follow-up, and diverse, scalable set of candidate moderators informing the PTR—improve the potential for generating findings that are both robust and implementable in real-world settings.

Beyond its methodological contributions, the findings from this study may offer practical insights for health system planning and resource allocation. Our cost-effectiveness analyses will assess whether optimizing treatment selection can improve efficiency by directing resources where they are most likely to help. By stratifying participants according to their predicted likelihood of remission, the findings could inform tiered implementation strategies that match intervention intensity to patient needs (92). Moreover, identifying probable non-responders to first-line treatment could support timelier referral to specialist care and improve continuity of services.

If successful, the findings from OptimizeD will demonstrate the feasibility of integrating precision approaches into routine mental health care in low-resource settings. It offers a unique opportunity to test whether data-driven treatment selection can both improve patient outcomes and make more efficient use of limited resources. Ultimately, the findings could potentially shift global mental healthcare toward a more adaptive, person-centered model that aligns treatment with individual needs.

## 3.1 Trial status

Trial recruitment started on 18 March 2024. Recruitment is tentatively scheduled to be completed in December 2025.

### Abbreviations

ADM – Antidepressant Medication

AIIMS – All India Institute of Medical Sciences

AMI – Attained Marginal Improvement

CATE – Conditional Average Treatment Effect

CNV – Copy Number Variation

C-SSRS – Columbia Suicide Severity Rating Scale

CSRI – Client Service Receipt Inventory

DSMB – Data and Safety Monitoring Board

GSA – Global Screening Array

GWAS – Genome-Wide Association Study

HAP – Healthy Activity Program

HS – High Sensitivity

IRB – Institutional Review Board

MOs – Medical Officers

MTI – Maximum Tolerated Imbalance

PHQ-9 – Patient Health Questionnaire-9

PRS – Polygenic Risk Score

PRS-CS – Polygenic Risk Score Continuous Shrinkage

PTR – Precision Treatment Rule

REDCap – Research Electronic Data Capture

SAE – Serious Adverse Event

SL – Super Learner

SNP – Single Nucleotide Polymorphism

SSRI – Selective Serotonin Reuptake Inhibitor

WHO – World Health Organization

WHODAS-II – World Health Organization Disability Assessment Schedule-II

## Declarations

*Ethics approval and consent to participate:* The study is approved by the ethics committees of Harvard Medical School (IRB20-2144, v11), Sangath (AB-2021-69, v1.7), and AIIMS Bhopal (EF0237). Additional approval, as required for all foreign-funded research in India, was obtained from the Government of India's Health Ministry Screening Committee, housed at the Indian Council of Medical Research. Amendments to the protocol are agreed upon by the TSC and approved by relevant IRBs prior to implementation. The consent process, conducted by trained and supervised Research Assistants, includes three stages: consent for the screening/eligibility survey, consent for main study enrollment for eligible participants, and a separate consent for blood or saliva sample collection. To standardize the process and ensure consistent delivery of key information, a video was developed to guide participants through the consent procedures. Written informed consent at each stage will be mandatory for enrolling in the trial. While study participants will not receive direct payment for their participation, they will receive 500 Indian Rupees (INRs) as reimbursement for the opportunity cost for each of the seven scheduled study assessments (baseline, week 1.5, week 6, week 12, month 6, month 9, and month 12), totaling 3,500 INR, approximately USD 40).

*Consent for publication*: Not applicable.

*Availability of data and dissemination*: Data generated and/or analyzed during the current study will be shared through the NIMH Data Archive (NDA) and can be accessed at https://nda.nih.gov/. To ensure participant privacy and data security, all shared data will be de-identified according to established protocols and guidelines. Researchers seeking access to the data must submit a formal data access request to the NIMH Data Archive and comply with data usage policies and guidelines. Data will be available indefinitely or for as long as the NDA repositories support it. Study findings will be published in open-access, peer-reviewed scientific journals and presented at conferences. We will also communicate the research findings to local stakeholders, including study participants, their families, and healthcare professionals, through community events at the clinics and informational materials such as fact sheets and posters.

*Conflict of interest:* The authors have nothing relevant to disclose.

*Funding:* This trial is funded by the National Institute of Mental Health (5R01MH121632-02), which underwent independent peer review as part of the funding approval process. The funder played no role in the study design, data collection, analysis, interpretation, manuscript writing, or submission for publication.

*Author's contributions*: JRP drafted the initial manuscript. All authors reviewed the manuscript and approved the final submission. Funding acquisition was led by VP, SDH, DRS, CL, PC, RDR, RCK, MH, AB, JAN, JWS, KC, AR, UK, AK, and TJV. All members of the Trial Steering Committee were involved in the methodology and implementation of the trial. VP and SDH provided oversight and guidance throughout the study and are co-senior authors.

*Acknowledgments:* We thank all the participants who generously took part in the study. We are also grateful to the state health system – the National Health Mission (NHM) Madhya Pradesh, the Mission Director (NHM Madhya Pradesh), the Chief Medical and Health Officer (CMHO) Bhopal, Assistant Program Manager (CMHO Bhopal office), Data Entry Operator (CMHO Bhopal office), the Medical Officers and other staff working in primary healthcare facilities, and the psychological treatment providers for their ongoing support. We also thank all the support staff across the collaborating institutions who enable the study to be conducted. Finally, we are grateful for the research staff whose dedication enables the daily implementation of the OptimizeD trial.

## Tables

Tables 1 to 4 are available in the Supplementary Files section.

## Figures

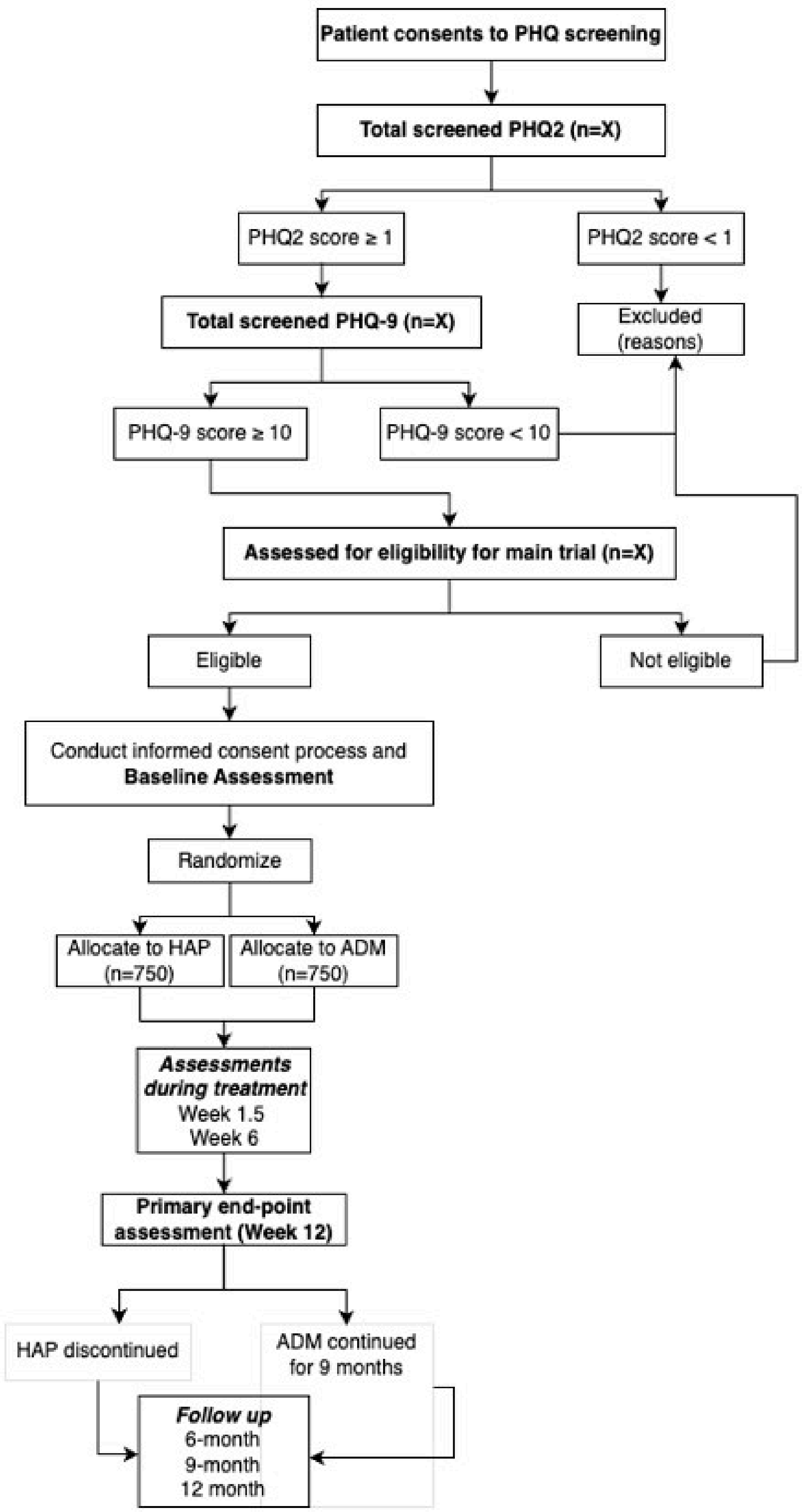

**Figure 1**

**Trial schema**

## Supplementary Files

This is a list of supplementary files associated with this preprint. Click to download.

- OptimizeDstudyprotocolappendix.docx
